# Mental Health Burden and Associated Factors Among Patients With Leprosy After Treatment Completion in South West Delhi

**DOI:** 10.7759/cureus.106685

**Published:** 2026-04-08

**Authors:** Suvigya Wadhwani, Nazish Rasheed, S K Rasania

**Affiliations:** 1 Community Medicine, Lady Hardinge Medical College and Associated Hospitals, New Delhi, IND

**Keywords:** activity limitations, anxiety, cross-sectional study, depression, impairments, india, leprosy, participation restrictions, post-mdt

## Abstract

Introduction

The psychological sequelae of leprosy extend beyond the course of disease, yet systematic mental health evaluation in post-treatment populations remains limited, particularly within urban Indian contexts. This study examined the prevalence and determinants of depression and anxiety among leprosy patients after treatment completion in South West Delhi.

Methods

A community-based cross-sectional study was conducted among 93 leprosy patients released from treatment between April 2021 and March 2024 in South West Delhi. The Patient Health Questionnaire-9 (PHQ-9) scale and the Generalized Anxiety Disorder-7 (GAD-7) scale were used to assess depression and anxiety, respectively. Impairment levels were graded by the WHO Disability Grading Classification, functional status through the Screening of Activity Limitation and Safety Awareness Scale, and the Participation Scale.

Results

The study population comprised 57 (61.3%) males and 36 (38.7%) females with a median age of 40 years. Most participants exhibited minimal psychiatric morbidity (depression: n = 73, 78.5 %; anxiety: n = 77, 82.8 %). Nevertheless, clinically meaningful depression (PHQ-9 ≥ 10) was seen in nine (9.7%) participants and clinically meaningful anxiety (GAD-7 ≥ 10) in seven (7.5%) participants. In multivariate linear regression, female gender (B = 1.70, 95% CI: 0.12 to 3.28, t = 2.14, p = 0.035); lower education levels: classes 9-12 (B = 2.80, 95% CI: 0.51 to 5.09, t = 2.43, p = 0.017), classes 1-8 (B = 2.32, 95% CI: 0.06 to 4.58, t = 2.04, p = 0.044), and no formal education (B = 2.78, 95% CI: 0.20 to 5.37, t = 2.14, p = 0.035); and participation restrictions (B = 5.97, 95% CI: 4.20 to 7.74, t = 6.70, p < 0.001) were significantly associated with higher depression scores (adjusted R²= 0.53, F = 11.27, p < 0.001), while just young age (< 40 years) (B = 1.51, 95% CI: 0.14 to 2.88, t = 2.19, p = 0.031) and participation restrictions (B = 4.23, 95% CI: 2.31 to 6.14, t = 4.40, p < 0.001) significantly predicted higher anxiety scores (adjusted R²= 0.31, F = 5.18, p < 0.001).

Conclusion

This study found that although most post-multi-drug therapy (MDT) leprosy patients reported minimal psychiatric morbidity, a notable proportion experienced clinically relevant depression and anxiety. Participation restrictions emerged as the strongest predictor of higher PHQ-9 and GAD-7 scores. Female gender, lower education, and activity limitations further increased depression risk, while younger age predicted higher anxiety. These findings underscore the need to integrate mental health services into the existing leprosy management protocols.

## Introduction

Leprosy, a chronic infectious disease caused by *Mycobacterium leprae*, primarily affects the skin and peripheral nerves, resulting in the classic hypopigmented, hypoesthetic lesions [1]. However, the consequences of leprosy extend far beyond its physical manifestations, with depression and anxiety rates higher than in the general population [2-4]. Despite achieving elimination as a public health problem in 2005, India contributes nearly 60% of the global annual leprosy cases, with 100,957 new leprosy cases detected in 2024 [5]. The National Leprosy Eradication Programme (NLEP), through accelerated case detection and treatment with multi-drug therapy (MDT), has achieved great success in leprosy control, substantially reducing the national leprosy prevalence rate from 57.2 per 10,000 population in 1981 to just 0.57 in 2025 [6].

Historically, leprosy has been associated with deep-rooted stigma, social ostracization, and economic deprivation [7,8]. Psychiatric morbidities, particularly depression and anxiety, represent a significant and often overlooked dimension of the leprosy burden. Previous studies consistently show a higher prevalence of psychiatric morbidities among people affected by leprosy (PAL), with depression rates reported between 12.6% and 53.5% across different global contexts [2-4,9]. The risk of psychiatric morbidities increases with the presence of visible impairments, loss of livelihoods, disruption of family roles, and community-level stigma [2-4]. While much of this evidence comes from rural or hospital-based studies of on-treatment patients, there remains a paucity of evidence from urban settings, particularly among patients who have completed MDT. South West Delhi, an urban metropolis characterized by an established healthcare system and a high literacy rate (88.3%) [10], offers a unique context to study the psychiatric burden in post-treatment PAL. Addressing this evidence gap is essential to developing effective post-treatment care frameworks that incorporate mental health support.

This study, therefore, had the following objectives: (1) to estimate the prevalence and pattern (distribution of severity levels) of depression and anxiety among leprosy patients after treatment completion in South West Delhi and (2) to examine the socio-demographic and clinical factors associated with depression and anxiety among leprosy patients after treatment completion in South West Delhi.

## Materials and methods

Study design, setting, and duration

This was a community-based cross-sectional study carried out in South West Delhi, India, from April 2024 to August 2025. The study area was urban, with a population of over 2.8 million and a literacy rate of 88.3% [10,11]. The Department of Community Medicine at Lady Hardinge Medical College and Associated Hospitals has field practice areas in two districts of Delhi: East Delhi and South West Delhi. South West Delhi was purposively selected based on OPD attendance data, demonstrating a higher leprosy patient load in its field practice area compared to the East Delhi field practice area during the study preparation phase.

Study population and sampling

Individuals aged ≥ 18 years, diagnosed with leprosy (new cases, both paucibacillary and multibacillary), who had completed the standard MDT and were officially “released from treatment (RFT)” between April 2021 and March 2024 according to the NLEP records, were included in the study. Exclusion criteria were (a) comorbid mental impairment precluding participation, (b) physical disabilities unrelated to leprosy, and (c) history of non-standard treatment. Of the 160 eligible RFT patients identified from NLEP records who met the inclusion criteria, 93 participants were recruited using proportionate stratified random sampling stratified by treatment release year (2021-2022: n = 16, 17.2%; 2022-2023: n = 27, 29.0%; 2023-2024: n = 50, 53.8%) to account for potential differences in treatment outcomes. From each yearly stratum, a random subset was drawn proportionally to its size using computer-generated random numbers, resulting in a total of 93 participants. Non-response or non-traceable cases (due to migration: n = 10; death: n = 2; inability to locate: n = 2; and refusal: n = 1) were replaced by randomly selecting participants from the same stratum in the sampling frame to meet the target sample size. Since this study is part of a thesis research project and was originally powered for WHO disability impairment, we performed a post-hoc sensitivity analysis (F test, linear multiple regression: fixed model, R² deviation from zero) with G*Power 3.1.9.7 (Heinrich-Heine-Universität Düsseldorf, Düsseldorf, Germany). Using the actual sample (N = 93) and the 10 predictors entered in the final multiple linear regression models, the analysis showed that with α = 0.05 and a desired power of 0.80, the smallest detectable effect size was f² = 0.19 (equivalent to R² = 0.16). Hence, our study was adequately powered to detect at least a medium effect size, corresponding to roughly 16% of the variance in the outcome measures.

Data collection and measurement tools

Comprehensive data were collected through face-to-face structured interviews in participants’ preferred language (Hindi), after obtaining written informed consent. Socio-demographic information was recorded, including age, gender, educational status, and other relevant background characteristics. Treatment history details were obtained, including leprosy type, detection delay, and the year of release from treatment. Impairment status was assessed using the WHO Disability Grading Classification, which categorizes physical impairments related to leprosy into grades 0, 1, and 2 [12]. Activity limitations were evaluated using the Screening of Activity Limitation and Safety Awareness (SALSA) scale and participation restrictions using the Participation Scale (P-Scale) [13-16]. For anxiety assessment, the Generalized Anxiety Disorder-7 (GAD-7), a seven-item validated instrument, was used (score range: 0-21). It classifies anxiety into minimal, mild, moderate, or severe, with scores ≥ 10 indicating moderate or higher anxiety levels (used for prevalence estimates) [17]. Depression was assessed using the Patient Health Questionnaire-9 (PHQ-9) tool, a nine-item validated tool, with scores ranging from 0 to 27, classifying depression into minimal, mild, moderate, moderately severe, and severe levels. Scores ≥ 10 indicate moderate or higher levels of depression (used for prevalence estimates) [18]. All questionnaires utilized are available in Hindi, validated, freely accessible, and require no formal permission.

Data analysis

Data were analyzed using IBM SPSS version 25 (IBM Corp., Armonk, NY). Descriptive statistics were used to summarize patient characteristics and mental health outcomes. To test associations between socio-demographic and clinical factors with depression and anxiety, we employed simple and multiple linear regression analyses rather than logistic regression, given the continuous nature of outcome measures (PHQ-9 and GAD-7 scores), to retain statistical power, and to avoid loss of information associated with categorization. Variables for multiple linear regression were selected if the association in simple linear regression was at p < 0.20 and based on known epidemiological importance. Normality of residuals was assessed using histograms and normal P-P plots of standard residuals; linearity and homoscedasticity were examined using scatter plots of standardized residuals versus standardized predicted values. Multicollinearity was assessed using variance inflation factors (VIF > 5). No significant violations were detected. There were no missing item-level data because questionnaires were checked in real time; thus, complete-case analysis was performed. Results are reported as unstandardized coefficients (B) with 95% confidence intervals. Two-tailed significance was set at α = 0.05.

Ethical considerations

The study protocol received ethical clearance from the Institutional Ethics Committee of Lady Hardinge Medical College before the commencement of data collection (vide letter no. (LHMC/IEC/2024/PG Thesis/14). Permission was obtained from the Delhi State Health Mission, Directorate General of Health Services (DGHS), Government of National Capital Territory of Delhi (vide letter no. (DSHM/NLEP/STUDY/01/45/2024-25/85-90)) to access patient databases and treatment registers. Study objectives, procedures, potential risks, and benefits were explained to all participants in their vernacular language, and written informed consent was obtained from all literate participants, while illiterate participants provided thumb impressions in the presence of literate witnesses. Interviews were conducted in a private setting to protect confidentiality, and data were anonymized using unique study codes with no personal identifiers in the analysis file. Participants reporting moderate-to-severe depressive or anxiety symptoms were counseled and referred to the nearest government health facility.

## Results

Socio-demographic and clinical profile

This study enrolled 93 individuals affected by leprosy in South West Delhi, comprising 57 (61.3%) males and 36 (38.7%) females. Of the study participants, 45 (48.4%) individuals were between 20 and 39 years of age (mean: 41.5 ± 13.9 years; range: 20-72; median: 40 years; IQR: 31-50.5 years), 37 (39.8%) had education beyond eighth grade, 38 (40.9%) were manual workers, and 35 (37.6%) were not working. Regarding leprosy classification, 86 (92.5%) participants were diagnosed with multibacillary leprosy, while seven (7.5%) had paucibacillary leprosy. Overall, 59 participants (63.4%) had no physical impairments, while 19 (20.4%) had grade 1 and 15 (16.1%) had grade 2 impairments. Regarding activity limitations, 24 (25.8%) participants had activity limitations, while 22 (23.7%) had participation restrictions (Table 1).

**Table 1:** Distribution of the study participants according to socio-demographic and clinical characteristics (N = 93) Values are presented as frequencies and column percentages (in parentheses). Percentages may not total 100% because of rounding.

Characteristic	Category	Male, n (%)	Female, n (%)	Total, n (%)
Age group	20-39 years	26 (45.6)	19 (52.8)	45 (48.4)
	40 years and above	31 (54.4)	17 (47.2)	48 (51.6)
Education	No formal education	12 (21.1)	11 (30.6)	23 (24.7)
	Classes 1-8	21 (36.8)	12 (33.3)	33 (35.5)
	Classes 9-12	18 (31.6)	10 (27.8)	28 (30.1)
	Graduate or above	6 (10.5)	3 (8.3)	9 (9.7)
Occupation	Manual workers	28 (49.1)	10 (27.8)	38 (40.9)
	Service workers	20 (35.1)	0 (0.0)	20 (21.5)
	Not working	9 (15.8)	26 (72.2)	35 (37.6)
Income tertile	Lowest	6 (10.5)	25 (69.4)	31 (33.3)
	Middle	21 (36.8)	9 (25.0)	30 (32.3)
	Highest	30 (52.6)	2 (5.6)	32 (34.4)
Marital status	Unmarried	5 (8.8)	6 (16.7)	11 (11.8)
	Married	45 (78.9)	26 (72.2)	71 (76.3)
	Separated/widowed	7 (12.3)	4 (11.1)	11 (11.8)
Leprosy classification	Multibacillary	53 (93.0)	33 (91.7)	86 (92.5)
	Paucibacillary	4 (7.0)	3 (8.3)	7 (7.5)
Detection delay	< 1 year	27 (47.4)	15 (41.7)	42 (45.2)
	≥ 1 year	30 (52.6)	21 (58.3)	51 (54.8)
WHO impairment grade	Grade 0	35 (61.4)	24 (66.7)	59 (63.4)
	Grade 1	11 (19.3)	8 (22.2)	19 (20.4)
	Grade 2	11 (19.3)	4 (11.1)	15 (16.1)
Activity limitations	Absent	40 (70.2)	29 (80.6)	69 (74.2)
	Present	17 (29.8)	7 (19.4)	24 (25.8)
Participation restrictions	Absent	43 (75.4)	28 (77.8)	71 (76.3)
	Present	14 (24.6)	8 (22.2)	22 (23.7)
Total		57	36	93

Prevalence and pattern of psychiatric morbidities

For depression, minimal symptoms were reported by 73 (78.5%) participants, mild symptoms by 11 (11.8%), moderate symptoms by seven (7.5%), and moderately severe and severe symptoms by one participant each (1.1%). In case of anxiety, 77 (82.8%) participants indicated minimal symptoms, nine (9.7%) had mild symptoms, five (5.4%) reported moderate symptoms, and two (2.1%) reported severe symptoms. Overall, clinically meaningful depression (PHQ-9 score ≥ 10) was observed in nine (9.7%) participants, while clinically meaningful anxiety (GAD-7 score ≥ 10) was seen in seven (7.5%) participants (Table 2).

**Table 2:** Distribution of depression and anxiety among study participants (N = 93) Values are presented as frequencies and column percentages (in parentheses).

Psychiatric morbidity	Severity	Total, n (%)
Depression level	Minimal	73 (78.5)
	Mild	11 (11.8)
	Moderate	7 (7.5)
	Moderately severe	1 (1.1)
	Severe	1 (1.1)
Anxiety level	Minimal	77 (82.8)
	Mild	9 (9.7)
	Moderate	5 (5.4)
	Severe	2 (2.1)
Total		93

In univariate regression analysis, participants younger than 40 years on average scored 1.71 points higher on the PHQ-9 (95% CI: 0.01 to 3.42, t = 2.00, p = 0.049) and 1.98 points higher on the GAD-7 (95% CI: 0.47 to 3.48, t = 2.61, p = 0.011). However, education and occupation both demonstrated clear trends of higher psychiatric scores with poor education and occupation outcomes; neither significantly predicted depression or anxiety. All three disability domains: physical impairments, activity limitations, and participation restrictions significantly predicted both mental health outcomes. Participation restrictions emerged as the strongest determinant of both depression and anxiety; participants reporting participation restrictions scored, on average, 6.72 points higher on the PHQ-9 (95% CI: 5.22 to 8.22, t = 8.91, p < 0.001) and 4.86 points higher on the GAD-7 scale (95% CI: 3.33 to 6.38, t = 6.32, p < 0.001). Likewise, participants with WHO impairments had 2.06 points higher depression scores (95% CI: 0.30 to 3.81, t = 2.33, p = 0.022) and 2.07 points higher anxiety scores (95% CI: 0.51 to 3.63, t = 2.64, p = 0.010), while those with activity limitations scored 4.55 points higher on the depression scale (95% CI: 2.81 to 6.30, t = 5.17, p < 0.001) and 2.92 points higher on the anxiety scale (95% CI: 1.25 to 4.60, t = 3.48, p = 0.001). The detailed univariate regression analyses for depression and anxiety are shown in Table 3 and Table 4, respectively.

**Table 3:** Univariate linear regression of socio-demographic and clinical factors with total mean PHQ-9 scores (N = 93) B = unstandardized regression coefficient; CI = confidence interval; PHQ-9 = Patient Health Questionnaire-9; SE = standard error; t = t statistic for B (B/SE); ß = standardized regression coefficient p < 0.05 is considered statistically significant.

Characteristic		PHQ-9 score					
		B	SE	ß	95% CI	t	p
Gender	Male	Reference					
	Female	0.89	0.90	0.10	-0.89-2.67	1.00	0.323
Age	≥ 40 years	Reference					
	< 40 years	1.71	0.86	0.21	0.01-3.42	2.00	0.049
Education	Graduate or above	Reference					
	Classes 9-12	1.97	1.61	0.22	-1.24-5.18	0.22	0.226
	Classes 1-8	2.02	1.58	0.23	-1.13-5.17	0.23	0.205
	No formal education	2.72	1.66	0.28	-0.57-6.01	0.28	0.104
Occupation	Service	Reference					
	Manual	0.22	1.17	0.03	-2.11-2.55	0.19	0.853
	Unemployed	0.35	1.19	0.04	-2.02-2.72	0.29	0.770
Income tertile	Upper	Reference					
	Middle	-0.1	1.07	-0.01	-2.23-2.04	-0.09	0.929
	Low	1.02	1.06	0.12	-1.10-3.13	0.96	0.341
Marital status	Married	Reference					
	Separated/widowed	1.07	1.37	0.08	-1.64-3.79	0.79	0.434
	Unmarried	1.53	1.37	0.12	-1.18-4.24	1.12	0.266
Type of leprosy	Paucibacillary	Reference					
	Multibacillary	2.02	1.65	0.13	-1.25-5.30	1.23	0.223
Detection delay	< 1 year	Reference					
	≥ 1 year	-0.06	0.88	-0.01	-1.81-1.69	-0.07	0.944
WHO impairment	Absent	Reference					
	Present	2.06	0.88	0.24	0.30-3.81	2.33	0.022
Activity limitations	Absent	Reference					
	Present	4.55	0.88	0.48	2.81-6.30	5.17	<0.001
Participation restrictions	Absent	Reference					
	Present	6.72	0.75	0.68	5.22-8.22	8.91	<0.001

**Table 4:** Univariate linear regression of socio-demographic and clinical factors with total mean GAD-7 scores (N = 93) B = unstandardized regression coefficient; CI = confidence interval; GAD-7 = Generalized Anxiety Disorder-7; SE = standard error; t = t statistic for B (B/SE); ß = standardized regression coefficient p < 0.05 is considered statistically significant.

Characteristic		GAD-7 score					
		B	SE	ß	95% CI	t	p
Gender	Male	Reference					
	Female	0.59	0.80	0.08	-1.00-2.19	0.74	0.461
Age	≥ 40 years	Reference					
	< 40 years	1.98	0.76	0.26	0.47-3.48	2.61	0.011
Education	Graduate or above	Reference					
	Classes 9-12	0.04	1.46	0.01	-2.85-2.94	0.03	0.976
	Classes 1-8	0.80	1.43	0.10	-2.04-3.64	0.56	0.578
	No formal education	0.70	1.50	0.08	-2.27-3.67	0.47	0.641
Occupation	Service	Reference					
	Manual	-0.16	1.05	-0.02	-2.24-1.92	-0.15	0.879
	Unemployed	-0.36	1.06	-0.05	-2.48-1.75	-0.34	0.733
Income tertile	Upper	Reference					
	Middle	-0.09	0.97	-0.01	-2.00-1.83	-0.09	0.928
	Low	0.26	0.96	0.03	-1.64-2.17	0.28	0.783
Marital status	Married	Reference					
	Separated/widowed	0.34	1.22	0.03	-2.09-2.76	0.28	0.784
	Unmarried	1.52	1.22	0.13	-0.91-3.94	1.24	0.217
Type of leprosy	Paucibacillary	Reference					
	Multibacillary	1.35	1.48	0.10	-1.59-4.29	0.91	0.364
Detection delay	< 1 year	Reference					
	≥ 1 year	0.71	0.78	0.10	-0.85-2.27	0.91	0.366
WHO impairment	Absent	Reference					
	Present	2.07	0.78	0.27	0.51-3.63	2.64	0.010
Activity limitations	Absent	Reference					
	Present	2.92	0.84	0.34	1.25-4.60	3.48	0.001
Participation restrictions	Absent	Reference					
	Present	4.86	0.77	0.55	3.33-6.38	6.32	<0.001

Multivariate linear regression analysis with the PHQ-9 explained 58% of the variance in PHQ-9 scores (adjusted R² = 0.53, F = 11.27, p < 0.001; Table 5). Female participants reported higher PHQ-9 scores than males (B = 1.70, 95% CI = 0.12 to 3.28, t = 2.14, p = 0.035). Age < 40 years was associated with a non-significant increase in PHQ-9 scores compared with participants ≥ 40 years (B = 1.22, 95% CI = -0.05 to 2.49, t = 1.91, p = 0.060). Lower education levels were also significant: classes 9-12 (B = 2.80, 95% CI: 0.51 to 5.09, t = 2.43, p = 0.017), classes 1-8 (B = 2.32, 95% CI = 0.06 to 4.58, t = 2.04, p = 0.044), and no formal education (B = 2.78, 95% CI: 0.20 to 5.37, t = 2.14, p = 0.035) had higher PHQ-9 scores compared with participants who had a graduate education. Manual workers had lower PHQ-9 scores than service-sector workers (B = -2.12, 95 % CI = -3.90 to -0.33, t = -2.36, p = 0.021); unemployment showed a nonsignificant trend toward lower scores (B = -1.95, 95 % CI = -4.02 to 0.13, t = -1.87, p = 0.065). Presence of activity limitations was associated with higher depressive scores (B = 2.22, 95% CI: 0.22 to 4.22, t = 2.21, p = 0.030), while participation restrictions remained the dominant predictor (B = 5.97, 95% CI: 4.20 to 7.74, t = 6.70, p < 0.001). Interestingly, WHO impairment was non-significant in the adjusted model (B = -0.78, 95 % CI = -2.24 to 0.69, t = -1.06, p = 0.294).

**Table 5:** Multivariate linear regression of socio-demographic and clinical factors with total mean PHQ-9 scores (N = 93) B = unstandardized regression coefficient; SE = standard error; ß = standardized regression coefficient; CI = confidence interval; t = t statistic for B (B/SE); p < 0.05 is considered statistically significant. R² = 0.58; adjusted R²  = 0.53; F = 11.27; p < 0.001 p < 0.05 is considered statistically significant.

Characteristic		PHQ-9 score					
		B	SE	ß	95% CI	t	p
Gender	Male	Reference					
	Female	1.70	0.80	0.20	0.12-3.28	2.14	0.035
Age	≥ 40 years	Reference					
	< 40 years	1.22	0.64	0.15	-0.05-2.49	1.91	0.060
Education	Graduate or above	Reference					
	Classes 9-12	2.80	1.15	0.31	0.51-5.09	2.43	0.017
	Classes 1-8	2.32	1.14	0.27	0.06-4.58	2.04	0.044
	No formal education	2.78	1.30	0.29	0.20-5.37	2.14	0.035
Occupation	Service	Reference					
	Manual	-2.12	0.90	-0.25	-3.90-0.33	-2.36	0.021
	Unemployed	-1.95	1.04	-0.23	-4.02-0.13	-1.87	0.065
WHO impairment	Absent	Reference					
	Present	-0.78	0.73	-0.09	-2.24-0.69	-1.06	0.294
Activity limitations	Absent	Reference					
	Present	2.22	1.00	0.23	0.22-4.22	2.21	0.030
Participation restrictions	Absent	Reference					
	Present	5.97	0.89	0.61	4.20-7.74	6.70	<0.001

The multivariate regression model for GAD-7 accounted for 39% of the variance in GAD-7 scores (adjusted R² = 0.31, F = 5.18, p < 0.001; Table 6). Participants less than 40 years of age reported higher anxiety levels than older participants (B = 1.51, 95% CI: 0.14 to 2.88, t = 2.19, p = 0.031). Female gender did not reach statistical significance (B = 1.31, 95 % CI = -0.39 to 3.02, t = 1.53, p = 0.129). Notably, education level, occupation, WHO impairment, and activity limitations were not significant predictors (all p > 0.05), whereas participation restrictions continued to exert the strongest effect (B = 4.23, 95% CI: 2.31 to 6.14, t = 4.40, p < 0.001).

**Table 6:** Multivariate linear regression of socio-demographic and clinical factors with total mean GAD-7 scores (N = 93) B = unstandardized regression coefficient; CI = confidence interval; GAD-7 = Generalized Anxiety Disorder-7; SE = standard error; t = t statistic for B (B/SE); ß = standardized regression coefficient R² = 0.39; adjusted R²  = 0.31; F = 5.18; p < 0.001 p < 0.05 is considered statistically significant.

Characteristic		GAD-7 score					
		B	SE	ß	95% CI	t	p
Gender	Male	Reference					
	Female	1.31	0.86	0.17	-0.39-3.02	1.53	0.129
Age	≥ 40 years	Reference					
	< 40 years	1.51	0.69	0.20	0.14-2.88	2.19	0.031
Education	Graduate or above	Reference					
	Classes 9-12	1.00	1.24	0.12	-1.47-3.47	0.80	0.425
	Classes 1-8	1.19	1.22	0.15	-1.24-3.63	0.98	0.332
	No formal education	1.45	1.40	0.17	-1.34-4.24	1.04	0.303
Occupation	Service	Reference					
	Manual	-1.66	0.97	-0.22	-3.58-0.26	-1.72	0.090
	Unemployed	-1.84	1.12	-0.24	-4.08-0.39	-1.64	0.104
WHO impairment	Absent	Reference					
	Present	0.26	0.79	0.03	-1.32-1.83	0.32	0.748
Activity limitations	Absent	Reference					
	Present	0.85	1.08	0.10	-1.31-3.00	0.78	0.437
Participation restrictions	Absent	Reference					
	Present	4.23	0.96	0.48	2.31-6.14	4.40	<0.001

## Discussion

This study assessed the socio-demographic and clinical correlates of depression and anxiety in an urban cohort of 93 post-treatment individuals affected by leprosy. Notably, our multivariate linear regression models explained 58% of the variance in PHQ-9 scores (adjusted R² = 0.53, F = 11.27, p < 0.001) and 39% of the variance in GAD-7 scores (adjusted R² = 0.31, F = 5.18, p < 0.001), highlighting a significant impact of socio-demographic and clinical factors on psychological outcomes in leprosy.

Socio-demographic profile

Our study population was relatively young (mean age of 41.5 ± 13.9 years). This age composition is similar to a multi-state Indian study, where 76% of participants were aged 18-50 years [3]. Likewise, a hospital-based study from Kolkata, India, reported a mean age of 44 years [2]. By contrast, studies from China and Nepal, in predominantly post-MDT cohorts, reported a mean age of 67.3 ± 13.1 years and 56.8 ± 13.54 years, respectively [4,19]. Our study's younger age profile is due to the inclusion of RFT cases from 2021-2024, comprising relatively recent cases compared to the Chinese and Nepalese cohorts [4,19]. Our study demonstrated a clear male predominance, with a male-to-female ratio of about 1.6:1. This male predominance is consistent with findings from multiple studies in India [2,3] and China [19]. However, studies from Nepal [4,9], Nigeria [20], and Brazil [21] have reported nearly equal gender distribution. The widespread pattern of male predominance across the literature reflects gendered barriers to care for women. The relatively higher educational profile of our study cohort, with 37 (39.8%) individuals reporting educational attainment above eighth grade, is consistent with the multi-state Indian study [3]. By contrast, studies from Nepal [4,9], Nigeria [20], and Bangladesh [22] have reported considerably lower educational attainment. The relatively better educational attainment in our study can be explained by higher literacy rates and better access to schooling in an urban metropolis.

Our study also highlights marked gender disparities, with 26 (72.2%) women not engaged in employment, compared to just nine (15.8%) men. Similarly, participants’ income levels demonstrate stark gender differences: 30 (52.6%) male participants were in the highest income group (> 14,000 Rs), whereas 25 (69.4%) female participants had no income. Similarly, in Brazil, a hospital-based study reported that more than half (55.3%) of study participants had family income ≤ one minimum wage [21]. The overwhelming evidence from this study and previous literature confirms that leprosy is deeply intertwined with poverty, acting as both a consequence of and a contributor to economic hardship. This study found that a large majority (n = 71, 76.3%) of participants were married, consistent with several other studies from India [2,3], Nepal [4,9], Brazil [21], and Nigeria [20].

Prevalence and distribution of depression and anxiety

In this urban community-based post-MDT cohort, the prevalence of clinically significant depression (n = 9, 9.7%) and anxiety (n = 7, 7.5%) was modest but still higher than general population estimates (4.7% depression and 3.0% anxiety) [23]. The prevalence of depression and anxiety in our study was considerably lower than that reported across multiple studies and geographical regions in the literature (11.8-69.3% depression and 10.2-51.3% anxiety rates, respectively) [2-4,9,19,20,24-26]. Studies that recruited participants from leprosy asylums or tertiary hospitals have reported markedly higher depression (22.5-53.5%) and anxiety rates (19-24.1%) [2,3,19]. Similarly, studies from settings where disability, low education, and economic dependence were common have reported higher rates of depression (24.6% in Nepal [4] and 53% in Bangladesh [26]) and anxiety (74% in Bangladesh) [26]. Exceptionally high depression (69.3%) and anxiety (51.3%) rates were seen in a mixed Neglected Tropical Disease (NTD) cohort (leprosy/Buruli ulcer patients) in Nigeria [20]. By contrast, our results closely resemble a Nepalese leprosy asylum cohort that reported a 12.6% depression and 10.1% anxiety rate using the Hospital Anxiety and Depression Scale (HADS) [9]. Our modest psychological burden reflects our relatively younger, educated, and low-disability cohort compared to less educated, older, high-disability, hospital- or asylum-based cohorts.

Socio-demographic correlates of depression and anxiety

In univariate analysis, gender was not significantly associated with either depressive (PHQ-9) or anxiety (GAD-7) scores. In the multivariate linear regression model, however, female gender emerged as an independent predictor of higher PHQ-9 scores (B = 1.70, 95 % CI 0.12 to 3.28, p = 0.035), while it remained non‑significant in the GAD-7 model (B = 1.31, p = 0.129). This pattern suggests that the crude association between gender and depression was initially masked by confounding. Closely resembling our findings, the multi-state Indian study highlighted female gender as a significant risk factor for higher PHQ-9 scores but not for GAD-7 scores [3]. In Ethiopia, women were 1.20 times more likely to have depressive symptoms and 1.34 times more likely to have anxiety symptoms than men [27]. Overall, our findings align with multiple studies documenting higher rates of depression and anxiety among women affected by leprosy, reinforcing the notion of triple burden for women with leprosy-gender disadvantage, disability, and stigma [3,4,21,26,27]. Our study found that younger age (< 40 years) was linked to higher PHQ-9 and GAD-7 scores; after adjustment, age remained significant for GAD-7 but not for PHQ-9. Available literature on age remains varied; for instance, research in Ethiopia has shown a higher risk of depression at older ages [27]. While our results align with those from China and India, where younger, economically active individuals faced higher distress [19,28]. Several other studies in India and Nepal have found no independent effect of age on psychological outcomes [3,4,9]. It is plausible that, in our relatively younger working-age urban cohort, younger people may be more concerned about employment, marriage, and future prospects, while older individuals might have developed coping mechanisms and accepted their condition.

Regarding education, our study found that lower education was associated with higher depressive scores and had no independent effect on anxiety scores. Likewise, in the multi-state Indian study, lower education levels were associated with depression and not with anxiety scores [3]. Research in China [19] and Nigeria [20] has reported higher odds of poor mental health with lower educational attainment.

In our cohort, manual occupation was associated with lower depressive scores compared with service occupations in multivariate analysis. This diverges from several studies where unemployment was a significant risk factor. Research from Kolkata observed that unemployment was associated with higher odds of depression in unadjusted analysis, although the effect attenuated in multivariable models [2]. In China, employment was associated with better mental health outcomes [19]; while in the mixed NTD cohort (leprosy/Buruli ulcer patients) from Nigeria, unemployment was associated with triple the odds of poor mental health [20]. Manual workers, such as those in construction or carpentry, engage in regular physical activity, a known protective factor against depression. In contrast, service-sector work often involves frequent social interaction, increasing fear of discrimination, and internalized stigma. Lower income levels and poor socio-economic status have been associated with a higher risk of poor mental health outcomes [3,21,25,26,28]. However, our analysis didn’t find any significant association between occupation and income levels and mental health outcomes.

Clinical and functional predictors

Notably, our study found that leprosy type and detection delay did not significantly predict psychiatric morbidities in our study population. In contrast, literature has frequently linked multibacillary leprosy and longer detection delays with higher risks of psychiatric morbidity [2,27]. In univariate regression analysis, each of the three disability domains - WHO impairment, activity limitations, and participation restrictions - was positively associated with higher PHQ-9 and GAD-7 scores (p < 0.05 for all). When all covariates (age, gender, education, occupation, and the three disability domains) were entered simultaneously, WHO impairment lost significance, suggesting that its relationship with mental-health outcomes was largely mediated by the functional limitations captured in the other two domains. By contrast, participation restrictions retained an independent and robust association and emerged as the strongest predictor of both depression (B = 5.97, 95% CI: 4.20 to 7.74, p < 0.001) and anxiety (B = 4.23, 95% CI: 2.31 to 6.14, p < 0.001). Activity limitations also remained a significant determinant of depressive symptoms (B = 2.22, 95 CI: 0.22 to 4.22, p = 0.03) but did not independently predict anxiety after adjustment. Research from Kolkata found that grade 1 or 2 disability quadrupled the odds of depression (adjusted OR 4.1), and moderate to extreme participation restrictions almost tripled the odds of depression (adjusted OR 3.1) [2]. A study from Nepal cited physical impairment as a strong independent predictor of poor mental health outcomes [4]. A study from China, using the Barthel index, significantly linked activity limitations with both depression and anxiety [25]. The overwhelming influence of participation restrictions in predicting poor mental health mirrors the shift from the purely biomedical model to the social model of disability, suggesting that physical impairments are most concerning when they translate into lost functions, roles, and social engagement.

Strengths and limitations

This study addresses a critical gap in leprosy research by simultaneously examining multiple dimensions of disability (physical impairments, activity limitations, and participation restrictions) and psychiatric morbidities among post-treatment PAL in an urban Indian setting. The systematic participant recruitment from the government database provides a representative community-based sample, avoiding the selection bias inherent in hospital-based or leprosy colony studies. Our statistical approach of testing model assumptions and the appropriate use of simple and multiple linear regression demonstrates statistical rigor. However, a cross-sectional study design can’t establish temporality, limiting causal inferences. The study was originally powered for WHO impairment rather than mental-health endpoints; although a post‑hoc sensitivity analysis suggests adequate power for medium effects in the regression models, smaller associations may not have been detected. Replacement of non-response or non-traceable cases might have introduced selection bias, as replaced participants could differ systematically from those originally selected, despite efforts to minimize bias through random selection and within-strata replacement. Furthermore, in a context where both leprosy and mental illness are stigmatized, the self-reporting nature of mental health outcomes, rather than clinician-confirmed psychiatric diagnoses, may have led to misclassification of cases and underestimation or overestimation of psychiatric morbidity. We also limited the number of predictors and did not include all available variables (e.g., income tertile, detection delay, leprosy type) in the multivariate model based on significance level in univariate analysis and epidemiological importance; however, this may have contributed to some residual confounding. Interaction or mediation analyses (e.g., examining whether participation restrictions mediate the relationship between impairment and depression) and mixed-methods approaches were beyond the scope and power of this study but are important directions for future research. Additionally, this was a single-center community-based study conducted in an urban setting, and the findings may not be generalizable to leprosy patients in rural areas or different health system contexts. Future multi-centric longitudinal studies with larger samples and appropriate control groups are required to confirm these findings, establish causality pathways, improve external validity, and allow comparison with mental health burden in the general population.

## Conclusions

The relatively low prevalence of clinically significant depression (9.7%) and anxiety (7.5%) in our study suggests that an urban context - with higher literacy, better healthcare access, and different socio-economic structures - provides some degree of protection against the psychological sequelae of leprosy, as observed more evidently in leprosy asylums, hospitals, or rural settings. Female gender, younger age (< 40 years), low education, activity limitations, and participation restrictions emerged as independent predictors of higher depressive scores, while manual occupation was protective against depression. Whereas only younger age (< 40 years) and participation restrictions were significantly associated with higher anxiety scores in our study. The strong influence of participation restrictions in our study emphasizes that functional limitations are more proximal predictors of mental ill health, rather than structural damage. However, several limitations, including the study's cross-sectional design, small sample size, and reliance on self-reported PHQ-9 and GAD-7 scores, temper the generalizability of these results. Consequently, while the data suggest that routine mental health screenings and tailored psychosocial support for younger people, women, and individuals with lower levels of education - particularly those with functional limitations - could mitigate the psychological consequences of leprosy, prospective, multicenter studies with larger cohorts are needed to confirm these associations and to evaluate the effectiveness of targeted psychosocial programs.
